# A vital sugar code for ricin toxicity

**DOI:** 10.1038/cr.2017.116

**Published:** 2017-09-19

**Authors:** Jasmin Taubenschmid, Johannes Stadlmann, Markus Jost, Tove Irene Klokk, Cory D Rillahan, Andreas Leibbrandt, Karl Mechtler, James C Paulson, Julian Jude, Johannes Zuber, Kirsten Sandvig, Ulrich Elling, Thorsten Marquardt, Christian Thiel, Christian Koerner, Josef M Penninger

**Affiliations:** 1 IMBA, Institute of Molecular Biotechnology of the Austrian Academy of Sciences, VBC – Vienna BioCenter Campus, Dr. Bohr-Gasse 3, 1030 Vienna, Austria; 2 Universitätsklinikum Heidelberg, Zentrum für Kinder- und Jugendmedizin, Analysezentrum III, Klinik für Kinderheilkunde I, Im Neuenheimer Feld 669, 69120 Heidelberg, Germany; 3 Department of Molecular Cell Biology and Centre for Cancer Biomedicine, Institute for Cancer Research, The Norwegian Radium Hospital, Oslo University Hospital, Montebello, 0379 Oslo, Norway; 4 Departments of Cell and Molecular Biology, Chemical Physiology, and Immunology and Microbial Science, The Scripps Research Institute, La Jolla, CA 92037, USA; 5 Institute of Molecular Pathology (IMP), Campus-Vienna-Biocenter 1, 1030 Vienna, Austria; 6 Department of Biosciences, University of Oslo, 0316 Oslo, Norway; 7 Universitätsklinikum Münster, Klinik für Kinderheilkunde, Albert-Schweitzer-Campus 1, 48149 Münster, Germany

**Keywords:** Ricin, toxin, fucosylation, sialylation, Lewis X, intracellular trafficking

## Abstract

Ricin is one of the most feared bioweapons in the world due to its extreme toxicity and easy access. Since no antidote exists, it is of paramount importance to identify the pathways underlying ricin toxicity. Here, we demonstrate that the Golgi GDP-fucose transporter Slc35c1 and fucosyltransferase Fut9 are key regulators of ricin toxicity. Genetic and pharmacological inhibition of fucosylation renders diverse cell types resistant to ricin via deregulated intracellular trafficking. Importantly, cells from a patient with SLC35C1 deficiency are also resistant to ricin. Mechanistically, we confirm that reduced fucosylation leads to increased sialylation of Lewis X structures and thus masking of ricin-binding sites. Inactivation of the sialyltransferase responsible for modifications of Lewis X (St3Gal4) increases the sensitivity of cells to ricin, whereas its overexpression renders cells more resistant to the toxin. Thus, we have provided unprecedented insights into an evolutionary conserved modular sugar code that can be manipulated to control ricin toxicity.

## Introduction

Ricin is a naturally occurring plant toxin produced in the seeds of *Ricinus communis*, the castor oil plant^1^. The toxin consists of two polypeptide chains, ricin toxin A chain (RTA) and ricin toxin B chain (RTB). Mediated by RTB with lectin properties, ricin binds to certain carbohydrate residues on the cell surface, and is internalized via retrograde transport into the Golgi and the ER. In the ER, the cytotoxic RTA dissociates and translocates across the ER membrane into the cytosol, where it irreversibly inactivates ribosomes^2^. Due to its potent toxicity and broad availability, ricin has been classified as a bioweapon^3^. Since no antidote exists and the toxin was found in the hands of extremists and terrorists, it is of paramount importance to uncover its mode of action and elucidate novel therapeutic avenues by identifying the pathways underlying ricin toxicity^4^. Although multiple cellular factors that are required for ricin toxicity have been identified, they often depend on cell type and organism of origin, as well as duration and amount of ricin exposure^5,6,7^.

β1,4 galactoses and N-acetylgalactosamine carbohydrate epitopes have been described as the major factors that ricin binds on the cell surface and mediate its toxicity. Shielding of terminal galactoses, e.g., through increased terminal sialylation, or the ablation of cellular galactosylation, results in a reduction of ricin binding and toxicity, consistent with ricin being a galactose-binding lectin^8,9,10^. Absence of fucosylation has also been suggested by us and others to increase resistance to the toxin^11,12^. Early reports have described that overexpression of an α1,3-fucosyltransferase in mutant hamster CHO cells makes them highly sensitive to ricin and other lectins^10,13^. In stark contrast to these observations, overexpression of a putative GDP-fucose transporter, Slc35c2, has been shown to suppress α1,3-fucosylation and increase resistance to ricin^11^. An explanation or further detailed analysis on these conflicting phenotypes has not been provided. Moreover, a potential interplay between galactosylation, fucosylation and sialylation has been implied in the context of ricin toxicity, but never studied in detail^14^. Thus, a thorough examination of the relevance and the exact role of fucosylation in ricin toxicity in different cell types and the underlying mechanistic principles are of paramount importance.

We here provide, for the first time, a detailed study of the role of the GDP-fucose transporter Slc35c1 and the α1,3-fucosyltransferase Fut9 in ricin toxicity, in diverse cellular backgrounds and human patient cells. The use of small-molecule inhibitors allowed us to study the effect of fucosylation, sialylation and their potential interplay, specifically in the context of the plant toxin. Our data led us to identify an evolutionary conserved druggable sugar code that can be manipulated to control ricin toxicity.

## Results

### Loss of Fut9 and Slc35c1 increases ricin resistance in different cell types

**Figure 1:**
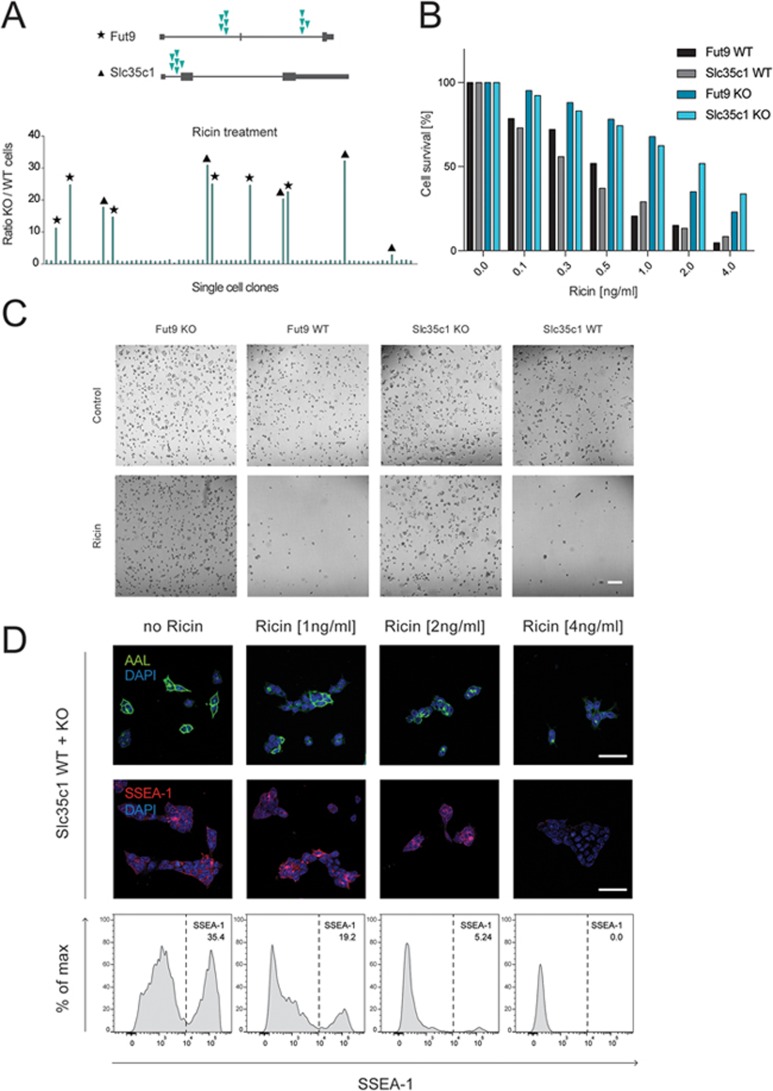
Loss of *Slc35c1* and *Fut9* protects cells from ricin toxicity. **(A)** Randomly mutagenized single-cell mESC clones were exposed to ricin (2 ng/ml) for 10 days and ratios of GFP+/mCherry+ cells were measured. Isolated, ricin-resistant, mutant clones were then analyzed via inverse PCR and their integration sites were determined. All clones were found to harbor the gene trap in sense orientation at the indicated intronic sites (green arrows) of either *Fut9* (asterisks) or *Slc35c1* (black triangles). **(B)** Survival of mESCs harboring a gene trap in either *Fut9* or *Slc35c1* in sense (KO) or antisense (WT) orientation in the presence of the indicated concentrations of ricin. Alamar Blue cell viability assay was used to determine cell survival. Data are representative of three independent experiments. **(C)** Independent *Fut9* and *Slc35c1* mutant (KO) and reverted WT mESC sister clones were grown in the presence or absence of ricin (8 ng/ml). Representative images are shown. Scale bar, 100 μm. **(D)** Mixed populations of unlabeled *Slc35c1* WT and mutant (KO) mESCs were exposed to different concentrations of ricin for 3 days. The amount of fucose (detected by AAL) and Lewis X (SSEA-1, CD15) expressing cells was monitored by immunofluorescence microscopy (upper panels) and flow cytometry (lower panels). Scale bar, 50 μm.

We and others previously identified Fut9 and Slc35c1 as candidate host factors required for ricin toxicity, without any further characterization or validation^11,12^. Both genes are involved in fucosylation: *Slc35c1* encodes a GDP-fucose transporter residing in the Golgi and *Fut9* encodes a Golgi α1,3-fucosyltransferase (Supplementary information, Figure S1A)^15,16^. To investigate potential roles of these genes and of fucosylation in ricin toxicity, we generated mouse embryonic stem cells (mESCs, haploid state) harboring a reversible gene trap in the first exon of *Fut9* or *Slc35c1* (Supplementary information, Figure S1B). Mutant clones harboring the gene trap in the sense orientation (knockout, KO) were GFP-positive (GFP+). Their respective wild-type (WT) sister clones, generated by infection with a virus encoding both mCherry and Cre recombinase, which reverses the gene trap and reconstitutes WT gene expression, were mCherry+. Loss of *Fut9* or *Slc35c1* in diploid murine ESCs did not affect embryonic stem cell identity, pluripotency (Supplementary information, Figure S1C), growth rates or survival, as indicated by constant ratios of GFP+/mCherry+ cells in culture. Upon treatment with ricin, however, multiple independently targeted *Fut9* and *Slc35c1* KO clones (GFP+) showed a survival advantage over reverted WT sister clones (mCherry+; Figure 1A and Supplementary information, Figure S2A). In line with previous findings^10,11^, *Fut9* and *Slc35c1* KO single-cell clones (diploid) showed an ∼10-fold increase in the LD_50_ of ricin compared to their WT sister clones (Figure 1B and 1C). A comparable phenotype of increased resistance was observed when we used the ricin homologue RCA_120_ (Supplementary information, Figure S2B).

Slc35c1 and Fut9 are required to generate the Lewis X epitope (SSEA-1, CD15; Supplementary information, Figure S1A), a prominent stem cell marker^17^. Indeed, *Fut9* and *Slc35c1* KO mESC clones lacked the fucose-containing SSEA-1 epitope on their cell surfaces (Supplementary information, Figure S2C). Loss of fucosylation was validated by reduced staining with *Aleuria aurantia* Lectin (AAL; Supplementary information, Figure S2D), which selectively binds fucose. Next, we generated mixed cell populations of *Slc35c1* (or *Fut9*) KO and repaired WT cells (diploid state), derived from the same parent clones, and monitored survival of SSEA-1-positive and SSEA-1-negative sister cells in response to ricin exposure. Loss of SSEA-1 conferred a selective survival advantage of cells in response to ricin treatment (Figure 1D and Supplementary information, Figure S2E). Thus, mESCs lacking SSEA-1, via loss of Fut9 or Slc35c1, are highly resistant to ricin.

**Figure 2:**
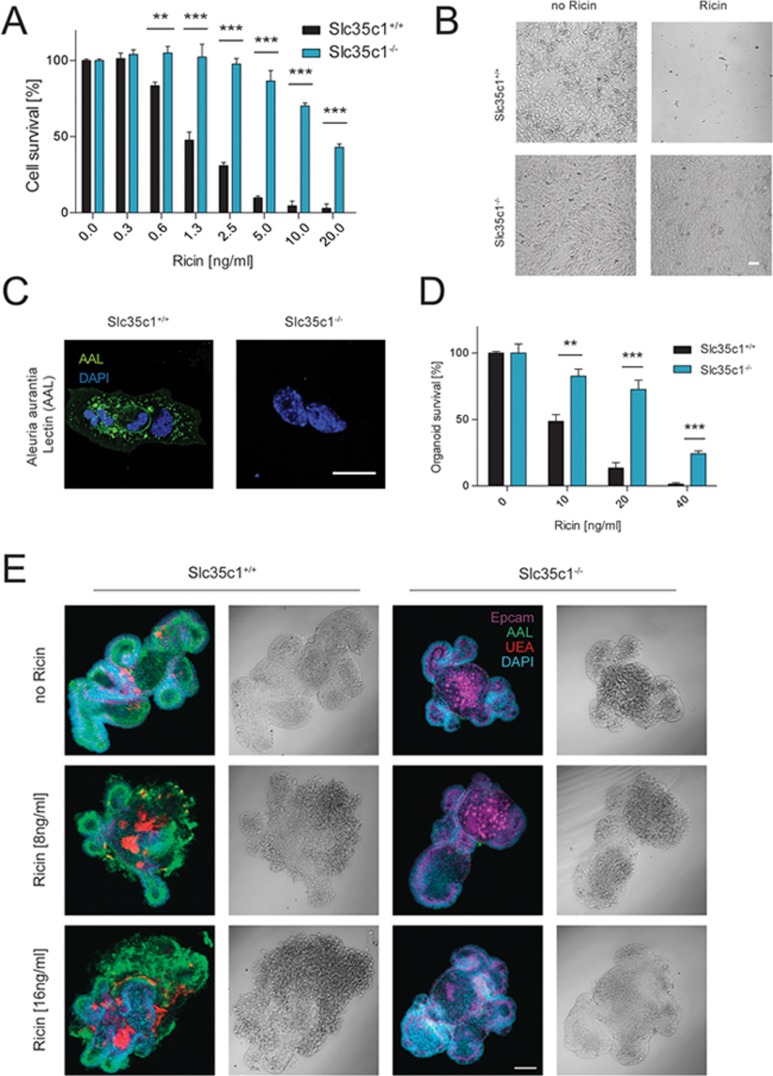
*Slc35c1* mutant MEFs and intestinal organoids show increased resistance to ricin. **(A)** *Slc35c1* WT and KO MEFs were cultured in the presence or absence of ricin. Cell survival was determined after 3 days by Alamar Blue. Data are shown as mean ± SD (*n* = 3) and are representative of three independent experiments. ^*^P < 0.05, ^**^P < 0.01, ^***^P < 0.001 (Student's *t*-test). **(B)** Representative images of *Slc35c1*^+/+^ and *Slc35c1*^−/−^ MEFs in the presence or absence of ricin (10 ng/ml) for 2 days. Scale bar, 50 μm. **(C)** WT and *Slc35c1* mutant MEFs were stained for the presence of fucose-containing glycans using AAL and microscopically analyzed. Scale bar, 25 μm. **(D)** Murine intestinal organoids isolated from control *Slc35c1*^+/+^ and mutant *Slc35c1*^−/−^ mice were treated with ricin (8 ng/ml) for 5 days and the numbers of surviving, intact organoids after ricin exposure were assessed. The percentage of surviving organoids compared to untreated gut organoids was determined. Data are shown as mean ± SD (*n* = 3). Representative data from five different experiments are shown. **(E)** *Slc35c1*^+/+^ and *Slc35c1*^−/−^ intestinal organoids were treated with different doses of ricin for 5 days and stained for markers (Epcam to detect epithelial cells; AAL to detect fucosylated glycans; and UEA to specifically detect α1,3-fucosylated glycan structures) to assess organoid integrity. DAPI was used as a nuclear counterstain. Representative images are shown. Scale bar, 50 μm.

To determine whether Slc35c1 influences ricin toxicity in other cell types, we evaluated how ricin treatment affected the survival of mouse embryonic fibroblasts (MEFs) isolated from *Slc35c1* WT (*Slc35c1*^+/+^) and KO (*Slc35c1*^−/−^) embryos^18^. Mutant cells harbor a disruptive neomycin resistance cassette in the first exon of the *Slc35c1* gene. Loss of Slc35c1 activity strongly protected MEFs from various concentrations of ricin, even at late time points (Figure 2A and 2B; Supplementary information, Figure S3A). Notably, *Slc35c1* KO MEFs completely lacked fucosylated structures (Figure 2C). As ricin ingestion can lead to accidental intoxication^19^, we investigated intestinal organoid cultures (mini-guts) generated from *Slc35c1* WT and KO mice (Supplementary information, Figure S3B). As expected, ricin treatment of WT organoids triggered pronounced morphological changes and loss of regenerative capacity compared to untreated controls. However, in the presence of ricin, *Slc35c1* KO organoids showed improved morphological integrity and increased survival compared to WT controls (Figure 2D and 2E; Supplementary information, Figure S3B). Moreover, splenocytes isolated from *Slc35c1* KO mice survived significantly higher doses of ricin than those from WT mice (Supplementary information, Figure S3C). Finally, *Slc35c1* homozygous KO mice that were exposed to sub-lethal dosages of ricin showed decreased weight loss compared to WT littermates (Supplementary information, Figure S3D). Thus, Slc35c1 plays a broad role in ricin toxicity.

### Mutation of human SLC35C1 confers resistance to ricin

**Figure 3:**
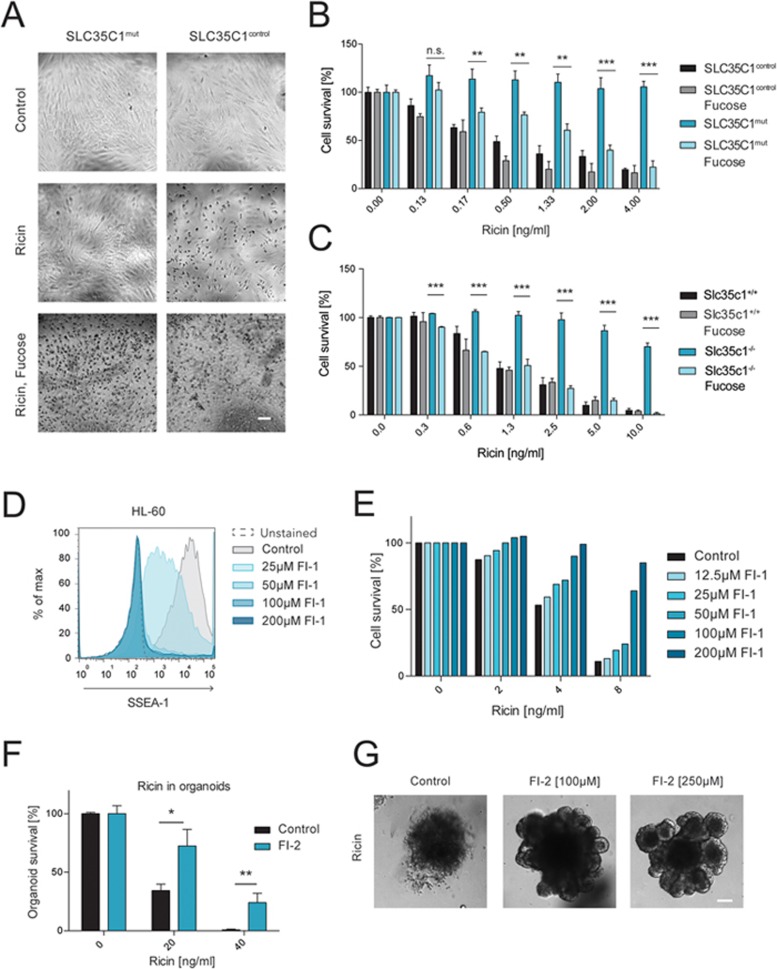
Loss or inhibition of fucosylation confers ricin resistance. **(A)** Control (*Slc35c1^control^*) as well as mutant (*Slc35c1^mut^*) HDFs were treated with ricin (4 ng/ml) and ℒ-fucose (10 mM) for 2 days. Their morphology and structural integrity were monitored by light microscopy. Scale bar, 50 μm. **(B)** Cell viability of *Slc35c1^mut^* and *Slc35c1^control^* HDFs, supplemented with ℒ-fucose (10 mM) for 24 h and treated with different doses of ricin for 48 h, was determined using Alamar Blue. Data are shown as mean ± SD of triplicate cultures. **(C)** Toxicity of ricin (after 48-h treatment) in *Slc35c1*^+/+^ and *Slc35^−/−^* MEFs cultured in the presence or absence of fucose (10 mM), was determined by Alamar Blue assay. Data are shown as mean ± SD of triplicate cultures. **(D)** Human HL-60 cells were treated with the indicated concentrations of the fucosylation inhibitor 2F-peracetyl fucose (FI-1) for 3 days, stained for SSEA-1 (CD15) and analyzed via flow cytometry. Normal SSEA-1 expression in HL-60 cells (control) as well as isotype-matched control cells (unstained) are shown. **(E)** HL-60 cells were pretreated with different concentrations of FI-1 and then exposed to different doses of ricin. Survival rates were assessed by Alamar Blue. Representative data of three independent experiments are shown. **(F)** The number of intact, surviving intestinal organoids was determined and the overall survival with and without the fucosylation inhibitor 2-deoxy-2-fluorofucose (FI-2, 250 μM) was assessed. Data are shown as mean ± SD of triplicate cultures and are representative for three different experiments showing similar results. **(G)** WT mouse intestinal organoids were pretreated with FI-2 and exposed to ricin (8 ng/ml) for 5 days. Representative images of organoids are shown. Scale bar, 50 μm.

Mouse and human glycosidic modification patterns can vary substantially^20^, therefore we investigated whether our observations in rodents extended to human cells. We assayed dermal fibroblasts isolated from a patient carrying a deficient *SLC35C1*^15^. Patients lacking SLC35C1 suffer from diverse symptoms, including severe immunological deficiencies; however, *SLC35C1* mutant human fibroblasts are virtually indistinguishable from control fibroblasts^15^. Upon treatment of these cells with ricin, control fibroblasts showed massive cell death, whereas *SLC35C1* mutant fibroblasts showed no morphological alterations and no signs of cell death (Figure 3A and 3B). Thus, genetic ablation of human *SLC35C1* confers resistance to ricin.

ℒ-Fucose administration to SLC35C1-CDG (congenital disorders of glycosylation) patients via their daily food intake reduces immunological and other clinical symptoms, potentially by allowing the utilization of the external source of sugar through a poorly characterized salvage pathway^21^. Remarkably, ℒ-fucose supplementation of *SLC35C1* mutant human fibroblasts not only reconstituted the expression of fucose, but also restored ricin sensitivity in these cells (Figure 3A and 3B; Supplementary information, Figure S3E). Similarly, *Slc35c1* KO MEFs acquired fucose-containing epitopes together with ricin sensitivity upon treatment with ℒ-fucose (Figure 3C and Supplementary information, Figure S3F). These results confirm a fucose-dependent role of Slc35c1 in ricin toxicity.

### Inhibition of cellular fucosylation is sufficient to confer ricin resistance

To test whether acute, pharmacological inhibition of fucosylation is sufficient to confer ricin resistance, we utilized 2F-peracetyl-fucose, a fucosyltransferase inhibitor that ablates fucosylation^22^. Treatment of the human cell line HL-60 with 2F-peracetyl-fucose led to a dose-dependent loss of fucose-containing Lewis X structures, as indicated by reduced SSEA-1 immunostaining (Figure 3D) and an increased resistance to ricin (Figure 3E). Furthermore, treating murine gut organoids with a similar pharmacological inhibitor of fucosylation (2-deoxy-2-fluorofucose)^23^ also increased resistance to ricin (Figure 3F and 3G). Thus, pharmacological inhibition of fucosylation is sufficient to confer ricin resistance.

### Slc35c1 is required for shuttling of ricin to the Golgi

**Figure 4:**
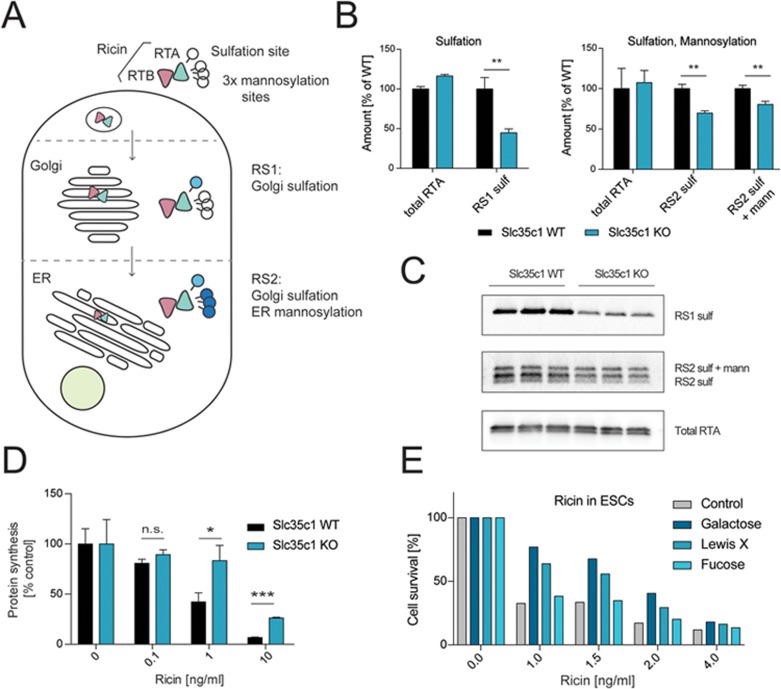
Fucosylation controls ricin toxicity via intracellular trafficking. **(A)** A modified version of the ricin toxin that harbors one specific sulfation (RS1) and three mannosylation (RS2) sites in the catalytically active RTA subunit allows for quantification of the amount of intracellular toxin trafficking to the Golgi and the ER, using radioactively-labeled isotopes. **(B**, **C)** *Slc35c1* WT and KO mESCs were incubated with engineered RTA (see scheme in **A**) to quantify ricin trafficking. The total amount of ricin (total RTA), as well as the amount of the sulfated, or sulfated and mannosylated form, was determined by western blot or autoradiography, respectively **(C)**, and quantified **(B)**. Data are shown as mean ± SD (*n* = 3). Each data set is representative of two independent experiments. ^*^P < 0.05, ^**^P < 0.01, ^***^P < 0.001 (Student's *t*-test). **(D)** The rate of protein synthesis was determined after 3 h of ricin exposure in *Slc35c1* control (WT) and *Slc35c1* mutant (KO) sister mESCs, using radioactively-labeled isotopes. Data are shown as mean ± SD of triplicate cultures. **(E)** Addition of galactose (200 μM) or Lewis X (200 μM), but not fucose (200 μM) increased viability of ricin-treated cells. Experiments were repeated three times; representative data are shown. NS, not significant; RTA, ricin toxin A subunit; RTB, ricin toxin B subunit.

To explore the mechanisms of ricin resistance, we tested whether cell surface binding or intracellular trafficking of ricin is altered in *Slc35c1*-deficient cells. While the RTB of ricin is essential for binding, internalization and trafficking, the catalytically active RTA can be modified to monitor ricin transport and quantify the amount of the modified toxin within different subcellular compartments (Figure 4A)^24^. Using this method, we did not observe a difference in ricin uptake between *Slc35c1* WT or KO cells, as determined by the total amount of intracellular RTA (Figure 4B and 4C). Loss of *Slc35c1* expression, however, markedly reduced the amount of ricin present in the Golgi and the ER, as indicated by the amount of sulfated and mannosylated RTA (Figure 4B and 4C). This suggests that fucosylation is required for targeted transport of ricin to the Golgi. Functionally, ricin-treated *Slc35c1* mutant cells displayed higher rates of protein synthesis than ricin-treated WT cells (Figure 4D), confirming reduced ricin-mediated ribosomal poisoning. Thus, loss of Slc35c1 expression does not affect binding and uptake of ricin, but impairs transfer from endosomes to the Golgi, and further to the ER, where ricin is ultimately processed for ribosomal poisoning.

### Fucosylation and sialylation play opposite roles in ricin toxicity

As Fut9 and Slc35c1 are required for the synthesis of both Lewis X (SSEA-1 positive) and sialyl Lewis X epitopes (SSEA-1 negative; Supplementary information, Figure S1A), we hypothesized that the protective effect of impaired fucosylation arises from a reduced display of β1,4 galactose-terminated Lewis X structures within host cells. We generated mutant mESCs that lack the β1,4-galactosyltransferase B4Galt1, which is involved in Lewis X biosynthesis. Upon treatment with ricin, *B4Galt1* KO (GFP+) mESCs showed markedly enhanced survival compared to their WT sister clones (mCherry+, Cre; Supplementary information, Figure S4A). This confirms that reduction of β1,4-galactosylation enhances resistance to ricin^10^. To test whether Lewis X itself is in principle susceptible to ricin binding, we added soluble, terminally galactosylated Lewis X tri-saccharide, galactose or fucose to cells and treated them with ricin. Addition of Lewis X or galactose (but not fucose) is sufficient to interfere with ricin toxicity and positively affect ESC survival in the presence of the toxin (Figure 4E).

**Figure 5:**
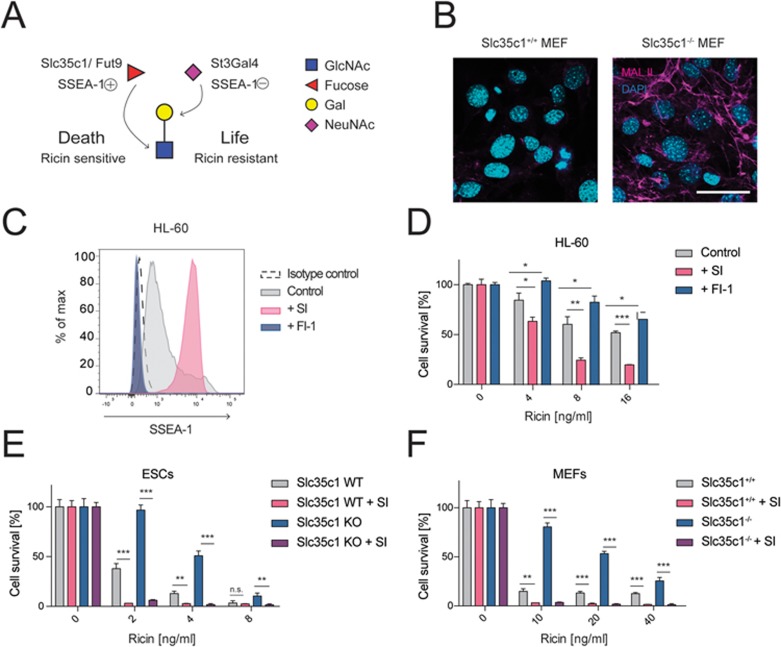
A vital sugar code for ricin toxicity. **(A)** Proposed sugar code for ricin toxicity. α1,3-fucose residues of Lewis X structures (SSEA-1^(+)^) impair α2,3-sialylation of terminal galactoses (i.e., sialyl Lewis X formation, SSEA-1^(−)^), leading to enhanced exposure of terminal galactoses and ricin binding. Absence of fucosylation allows more efficient sialylation of terminal galactoses and thus is assumed to inhibit ricin binding. GlcNAc (N-acetylglucosamine), Gal (Galactose), NeuNAc (N-Acetylneuraminic acid, one type of sialic acid). **(B)** *Slc35c1* mutant and WT MEFs were stained for α2,3-sialic acid using MALII and counterstained with DAPI to image nuclei. Scale bar, 50 μm. **(C)** HL-60 cells were treated with the fucosylation inhibitor 2F-peracetyl fucose (FI-1, 100 μM) or the sialylation inhibitor 3F_ax_-peracetyl Neu5Ac (SI, 250 μM) for 3 days. The amount of the fucose containing, un-sialylated epitope SSEA-1 (CD15) was determined via flow cytometry. SSEA-1 expression of vehicle-treated cells (control) as well as an isotype-matched control (isotype control) is shown. **(D)** HL-60 cells were pretreated with inhibitors of fucosylation (FI-1, 100 μM) or sialylation (SI, 250 μM) and exposed to different amounts of ricin thereafter. The survival of the cells was determined using Alamar Blue. Data are representative of three independent experiments. **(E**, **F)** *Slc35c1* wild type (WT) and mutant (KO) mESCs **(E)** and MEFs **(F)** were treated with SI (250 μM) and their sensitivity to ricin was assessed using Alamar Blue. Data in **D**-**F** are shown as mean ± SD of triplicate cultures. Experiments were repeated three times with similar results. ^*^P < 0.05, ^**^P < 0.01, ^***^P < 0.001; NS, not significant (Student's *t*-test).

Previous studies suggested that fucosylation impedes the extent of sialylation of Lewis X and related structures^25^ (Supplementary information, Figure S1A). Thus, we reasoned that fucosylation promotes the exposure of otherwise masked (i.e., sialylated) terminal galactose residues in WT cells. In contrast, *Slc35c1* KO cells would lack fucosylation and thus all terminal galactoses could be sialylated, resulting in resistance to ricin. In line with previous findings^8^, our model predicts that inhibiting sialylation in cells would not only lead to increased amounts of Lewis X (SSEA-1 positive), but also deprive terminal galactoses of sialic acids^9,14^ and thus increase sensitivity to ricin (Figure 5A).

In concordance with this model, genetic inactivation of fucosylation led to increased terminal sialylation of galactoses (Figure 5A and 5B)^26^. Next, we treated HL-60 cells with the sialylation inhibitor 3F_ax_-Peracetyl Neu5Ac^22^ and observed an increased expression of the SSEA-1 epitope (Figure 5C) as well as an increased sensitivity to ricin (Figure 5D). In addition, 3F_ax_-Peracetyl Neu5Ac-treated human dermal fibroblasts (HDF), canine MDCK cells and monkey Vero cells also displayed increased sensitivity to ricin (Supplementary information, Figure S4B-S4D). Further, increased resistance of *Slc35c1* KO mESCs (Figure 5E) and MEFs (Figure 5F) was abolished upon inhibition of sialylation. Thus, loss of sialylation increases ricin sensitivity.

### Ablation of the α2,3-sialyltransferase St3Gal4 increases susceptibility to ricin

**Figure 6:**
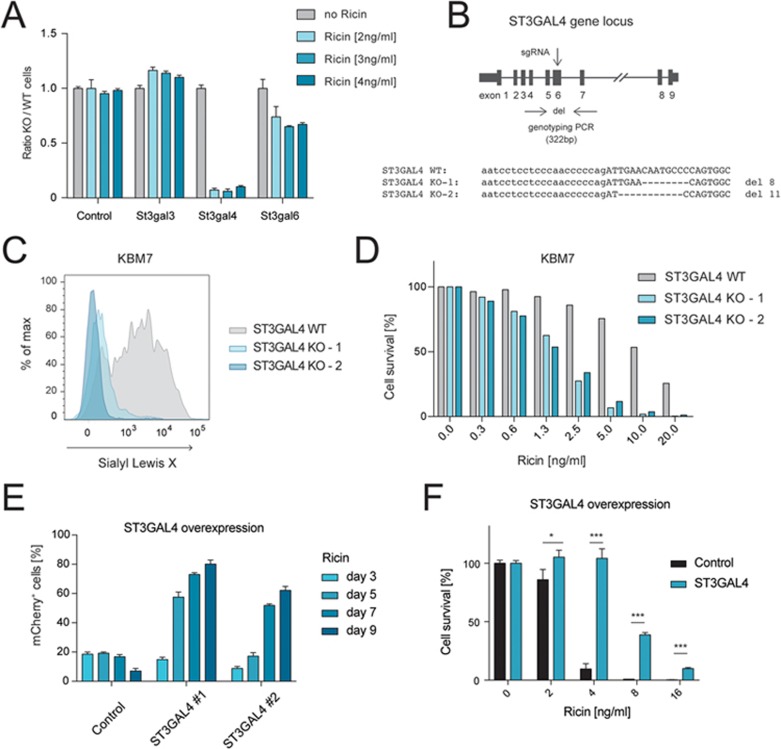
St3Gal4 determines susceptibility or resistance to ricin. **(A)** Mixed populations of cells that harbor a reversible gene trap in *St3Gal3*, *St3Gal4* or *St3Gal6*, as well as parental WT control cells, were subjected to ricin treatment for 3 days. The ratio of mutant (sense, GFP) to WT (antisense, mCherry/Cre) cells was assessed via flow cytometry. Data are shown as mean ± SD of triplicate cultures. **(B)** Human near haploid KBM7 cells were used to generate *ST3GAL4* KO clones using CRISPR/Cas9. Genomic PCR and sequencing of mutagenized clones, as well as control cells, showed appropriately targeted loci (8-bp or 11-bp deletions). Exons are indicated as black boxes. The guide RNAs were designed to delete sequences in exon 6 (arrow). Two different mutant clones were generated (ST3GAL4 KO-1, KO-2). **(C)** ST3GAL4 mutant and control human KBM7 cells were stained for sialyl Lewis X (CD15s) and analyzed via flow cytometry. **(D)** ST3GAL4 KO-1 and KO-2 KBM7 cells, as well as control cells, were treated with different amounts of ricin and their viabilities were determined. Data are representative of three independent experiments. **(E**, **F)** mESCs were infected with a doxycycline-inducible expression construct coding for *ST3GAL4* together with mCherry, or an empty control vector coding for mCherry only. **(E)** Mixed populations of infected and uninfected, as well as control cells, were treated with doxycycline and exposed to ricin (4 ng/ml) for 9 days. The percentage of mCherry+ cells was monitored over time by flow cytometry. Data are shown as mean ± SD of triplicate cultures. **(F)** An ST3GAL4-overexpressing mESC clone as well as an empty vector control were treated with various concentrations of ricin and their viabilities were determined using Alamar Blue staining. Data are shown as mean ± SD of triplicate cultures.

To uncover the sialyltransferase that is required for ricin resistance, we generated mESC mutants and their respective repaired sister clones for the three potential α2,3-sialyltransferase candidate genes, namely *St3Gal3*, *St3Gal4* and *St3Gal6*. These enzymes can catalyze the generation of sialyl Lewis X (Supplementary information, Figure S1A), but differ in their expression patterns and catalytic efficiencies^27^. We exposed mixed populations of mutant and repaired, WT mESCs (diploid state) to ricin and observed increased ricin sensitivity in cells lacking *St3Gal4* (Figure 6A), similar to previous reports^28,29^. As expected, ricin sensitivity correlated with a reduced level of sialyl Lewis X in these cells (Supplementary information, Figure S5A). No or minor changes, respectively, were observed in *St3Gal3* and *St3Gal6* mutants (Figure 6A and Supplementary information, Figure S5A). *St3Gal4* mutant mESCs were also more sensitive to RCA_120_ (Supplementary information, Figure S5B).

To confirm our findings in human cells, we used CRISPR/Cas9 to disrupt *ST3GAL4* in the near haploid leukemic cell line KBM7^30^. We identified two independently targeted single-cell clones that showed a partial deletion of exon 6 at the *ST3GAL4* genomic locus (Figure 6B) and confirmed loss of sialyl Lewis X in these two KO cell lines compared to control cells (Figure 6C). Both *ST3GAL4*-mutated KBM7 clones showed higher Lewis X levels (Supplementary information, Figure S5C) as well as markedly increased sensitivity to ricin relative to the control (Figure 6D). To further explore the role of α2,3-sialylation and St3Gal4 in ricin toxicity, we generated mESC with a doxycycline-inducible *ST3GAL4* gene linked to mCherry. Mixed populations of infected and uninfected cells, as well as vehicle-treated control cells, were treated with doxycycline and ricin or RCA_120_. A selective enrichment of cells that expressed ST3GAL4-mCherry and lacked SSEA-1 could be observed over time (Figure 6E; Supplementary information, Figure S5D and S5E). These results were confirmed in isolated mESC clones that overexpress ST3GAL4 (Figure 6F). Thus, St3Gal4 is indeed the key sialyltransferase involved in ricin toxicity in mESCs, and loss of *St3Gal4* renders mESCs hypersensitive to ricin killing.

## Discussion

The plant toxin ricin is one of the most poisonous naturally occurring substances known^1^. Ricin has been researched and used as an agent of biological warfare for more than 100 years, and has recently been found in the hands of terror organizations^3^. Here, we firmly establish that fucosylation is crucial for ricin toxicity through its inhibitory effects on cellular sialylation patterns, specifically in the context of Lewis X. Ablation of the GDP-fucose transporter Slc35c1 or inhibition of cellular fucosylation is sufficient to increase resistance to the toxin in different cell types. Absence of sialylation, or more specifically inactivation of α2,3-sialyl transferase St3Gal4, increases sensitivity to ricin and is sufficient to reverse resistance. Of note, absence of fucosylation does not affect binding or uptake of the toxin, but specifically decreases the amount of ricin that is shuttled to the Golgi and the ER. Our data corroborate that only a small (5%) fraction of ricin that is transported to the Golgi^31^ accounts for ricin toxicity. We hypothesize that although ricin binds to a plethora of galactose displaying proteins or lipids on the cell surface, only a specific fraction of those factors is internalized into a cell and can mediate trafficking of ricin into endosomes or the Golgi. A more detailed understanding of the specific, fucosylation-dependent targets that ricin binds to and orchestrate intracellular shuttling of the toxin, is required.

Our data for the first time identifies an exquisite specificity of ricin for defined glycosidic structures that determine cellular fate upon exposure to the toxin. Although protein glycosylation vastly differs between species^20^, we reveal that this vital sugar code for ricin toxicity is conserved from mouse to human and acts as an essential and evolutionary conserved switch between sensitivity and resistance to the toxin. Drugs affecting this sugar code impact the survival of cells exposed to ricin, and may be of therapeutic relevance for this bioweapon.

## Materials and Methods

### Cell lines and cell culture

mESCs (clone AN3-12)^12^ were cultured in DMEM supplemented with 10% fetal bovine serum (FBS), penicillin-streptomycin, non-essential amino acids, sodium pyruvate (1 mM), ℒ-glutamine (2 mM), β-mercaptoethanol (100 μM) and LIF (20 μg/ml). HDFs, MEFs, Vero cells, MDCK cells, HEK293 cells and PlatE cells were cultured in DMEM supplemented with 10% fetal calf serum (FCS), penicillin-streptomycin and ℒ-glutamine. HL-60 and KBM7 cells were cultured in IMDM supplemented with 10% FCS, penicillin-streptomycin and ℒ-glutamine. Haploid murine ESCs were used for insertional mutagenesis and derivation of gene trap-harboring KO cell lines as described previously^12^. Near haploid human KBM7 cells were utilized for CRISPR/Cas9-mediated KO generation. PlatE and HEK293 cells were used for recombinant retrovirus and lentivirus production.

### Insertional mutagenesis in murine ESCs

Haploid murine ESCs were generated previously^1^ and used for reversible mutagenesis (i.e., derivation of gene trap-harboring KO and genetically repaired sister cell lines). mESCs harboring a gene trap in introns of *Slc35c1* (chr2:92457575, GRCm38/mm10) and *Fut9* (chr4:25795404, GRCm38/mm10), as well as *B4Galt1* (chr4:40843768, GRCm38/mm10), *St3Gal3* (chr4:118076419, GRCm38/mm10), *St3Gal4* (chr9:35110455, GRCm38/mm10) and *St3Gal6* (chr16:58511873, GRCm38/mm10) were generated as described^1^. For the reversion of the gene trap, clones were infected with retroviruses carrying mCherry together with Cre recombinase. Retroviruses carrying GFP alone were used to label KO cells. For single-cell clones, cells were transiently transfected with a plasmid encoding Cre recombinase as well as GFP, and single-cell clones were sorted and then analyzed using PCR. The orientation of the splice acceptor was determined using a three-primer-containing amplification system, consisting of a fragment binding 1st forward primer or the inverse forward primer. In general, haploid ESCs were used for mutagenesis and the generation of homozygous KO cell lines; subsequently, these cells diploidize via endo-replication and hence carry the mutation at both alleles; these cells were used for all experiments. The following set of primers was applied (5′-3′):

GT 1st Fwd TCGACCTCGAGTACCACCACACT

GT inverse Fwd AAACGACGGGATCCGCCATGTCA

GT common Rev TATCCAGCCCTCACTCCTTCTCT

### CRISPR/Cas9 mutagenesis

Human *ST3GAL4* exon 6 in near haploid KBM7 cells was mutated using a lentiviral expression vector encoding the respective sgRNA (sgRNA: fwd 5′-CACCGATTGAACAATGCCCCAG-3′, rev 5′-AAACCTGGGGCATTGTTCAATC-3′) as well as Cas9, together with a GFP marker gene. After infection with the vector, GFP-positive cells were sorted via FACS and single-cell clones were generated. Control as well as mutated genomic loci of engineered cell lines were Sanger sequenced and the integrity of the respective loci was assessed.

### Generation of overexpression cells

Human *ST3GAL4* cDNA was amplified using the following primers: 5′-gttggatccaccATGTGTCCTGCAGGCTGGA-3′, and 5′-gcggaattcgtcgacTCAGAAGGACGTGAGGTTCTTG-3′, and cloned into a doxycyclin-inducible retroviral expression vector using either *Bam*HI or *Eco*RI similar to previous reports^32^. Insertion of the vector was monitored using Venus fluorophore; induction of gene expression following doxycycline treatment was examined by mCherry expression.

### Competitive growth assays

Diploid murine ESCs harboring a gene trap in introns of the respective genes were seeded at low density in normal ESC growth medium and infected for 12 h with two viruses, one encoding mCherry plus Cre recombinase and the other coding for GFP, both together with puromycin (Invivogen, ant-pr-1). Infected cells were selected (final concentration of puromycin, 1 μg/ml) after 24 h and expanded. Ratios of GFP- to mCherry/Cre-expressing cells in the presence and absence of ricin were determined using high-throughput flow cytometry (BD LSRFortessa HTS cell analyzer).

### Immunofluorescence and immunocytometry

Paraformaldehyde (4%) fixed cells were blocked and permeabilized for 1 h at room temperature with 1× PBS supplemented with 0.2% Triton, 1% glycine, 5% FBS and 2% bovine serum albumin (BSA). Specimens were incubated with the following primary antibodies: anti-Oct-3/4 (Clone 40/Oct-3 (RUO), BD Transduction Laboratories, #611203, 1:300), anti-SSEA-1 (CD15, Lewis X; PE conjugated, clone: MC-480, eBioscience 12-8813-41, 1:300), Epcam (Alexa Fluor 647 anti-mouse CD326 (Ep-CAM), Biozym, B118212, 1:400) or lectins: AAL (biotinylated, Vector Labs B1395, 1:500), Maackia Amurensis Lectin II (MALII, biotinylated, Vector Labs B1265, 1:300), Ulex Europaeus Agglutinin (UEA, Rhodamine labeled, Vector Labs RL-1062, 1:400). For the stainings shown in our study, cells were incubated with antibodies or lectins overnight at 4 °C, washed 3 times with 1× PBS supplemented with 0.2% Triton and 1% glycine, and then incubated with secondary reagents (Streptavidin, Alexa Fluor 633 conjugate, Invitrogen, S21375, 1:500) at room temperature for 1 h. Images were acquired with a laser scanning confocal microscope (LSM780 Axio Observer, Carl Zeiss) and analyzed using Zeiss imaging software. For flow cytometry staining, cells were trypsinized and incubated with murine or human antibodies to Lewis X (clone as above), CD15s (sialyl Lewis X; clone CSLEX1, Becton Dickinson, 551344, 1:500) in 1× PBS supplemented with 5% BSA. Primary or biotinylated antibodies were detected with secondary reagents (F(ab')2 anti-mouse IgG APC, eBioscience 17-4010-82, 1:500; Streptavidin PE-Cy7, Biolegend 405206, 1:1 000). Samples were analyzed using flow cytometry (LSR Fortessa, BD) or FACS-sorted using an Aria III flow cytometer (BD). Alkaline phosphatase staining was performed according to the manufacturer's protocols (VECTOR Blue Alkaline Phosphatase (Blue AP) Substrate Kit, Catalog#: SK-5300).

### Ricin toxicity assays

Ricin crude extracts in PBS were generated as described^33^. To assess ricin toxicity in different cellular systems, cells were plated at low density (5-10 cells/well in 96-well plates) in growth medium. Different concentrations of ricin were added to the cells upon seeding for at least 24 h in triplicate cultures. The cell viability at different time points was determined using the Alamar Blue Cell viability assay (according to the manufacturer's protocols) or automated quantification of viable cells using flow cytometry (BD LSRFortessa cell analyzer). Both types of analysis showed comparable results and were thereafter used interchangeably for most cell types. Cell death was confirmed using multiple other assays such as Fixable Viability Dye eFluor 780 (FVD eFluor 780, Affimetrix Biosciences, 65-0865-14) and DAPI (4,6-diamidino-2-phenylindole, dilactate; Invitrogen, D357) stainings.

### Intracellular ricin toxin trafficking

For detection of sulfation, cells were washed twice in sulfate-free medium supplemented with 2 mM ℒ-glutamine, followed by incubation with 0.2 mCi/ml H235SO4 in sulfate-free medium for 2.5 h at 37 °C. The cells were then incubated with ricin-sulf-1 (RS1, 6 μg/ml) or ricin-sulf-2 (RS2, 6 μg/ml) for an additional 2 h. RS1 and RS2 proteins have been described previously^34^. The cells were then washed twice for 5 min with 0.1 M lactose in HEPES-buffered medium, and once in ice-cold PBS before addition of 400 μl lysis buffer (0.1 M NaCl, 10 mM Na_2_HPO_4_, 1 mM EDTA, 1% Triton X-100, 60 mM octyl glucopyranoside) supplemented with complete protease inhibitors (Roche Diagnostics, Mannheim, Germany). Cell lysates were centrifuged for 10 min at 6 000 rpm at 4 °C to remove the nuclear fraction. Toxin constructs were immunoprecipitated overnight at 4 °C using protein A sepharose beads (Amersham Biosciences, Buckinghamshire, UK), coated with rabbit anti-ricin antibody (Sigma-Aldrich, R1254). Beads were washed twice in 0.35% Triton X-100/1× PBS and re-suspended in sample buffer. Proteins were separated on a SDS-PAGE gel, blotted onto a PVDF membrane (Immobilon-P, Millipore, Billerica, MA, USA) and analyzed by autoradiography using the PharosFX scanner and Quantity One 1-D Analysis Software 4.6.5 (Bio-Rad Laboratories, CA, USA). The amount of mannosylated RS2 was determined from the upper band of the RS2 sulfation blot, representing RS2 containing both sulfation and mannosylation modifications. The total amount of sulfated proteins was determined by trichloroacetic acid (TCA) precipitation of all ^35^S-labeled proteins in the lysates, and the total amount of internalized ricin (total RTA) was determined by western blot, using an anti-RTA antibody (clone CP75, Abcam, ab20968, 1:1 000).

### Protein synthesis

Cells grown in 24-well plates were incubated with increasing concentrations of ricin (0, 1-100 ng/ml) for 3 h at 37 °C. The medium was then replaced with leucine-free HEPES-buffered medium containing 2 μCi/ml [^3^H]Leucine and the cells were incubated further for 20 min. Proteins were precipitated with 5% (w/v) TCA, washed once in 5% (w/v) TCA, and dissolved in 0.1 M KOH. The incorporation of radioactively-labeled leucine was quantified, and IC_50_ was calculated as the concentration of toxin giving a 50% reduction in protein synthesis. Fold protection was then calculated as the ratio between IC_50_ for inhibitor-treated cells compared to control cells.

### Mouse experiments

*Slc35c1* KO mice have been previously described^18^. Genotypes were assessed by PCR. Only age- and sex-matched littermates (male) from respective breedings were used for experiments. Mice were injected intravenously with a sub-lethal dose of ricin (0, 53 μg/kg). The body weight of mice before the treatment was determined, and the weight loss over the indicated period of time was monitored. Mice were maintained in temperature-controlled conditions. Of note, we gave sub-lethal doses of ricin because death experiments as an end point are not allowed, due to ethical regulations and new Austrian legislature. All animal experiments were carried out in agreement with the ethical animal license protocol in accordance with the current laws of Austria.

### Intestinal organoid cultures

Intestinal organoid cultures were isolated and cultivated as described previously^35^. In brief, intestines were isolated from *Slc35c1*^+/+^ and littermate *Slc35c1^−/−^* mice, flushed with PBSO medium (Ca-Mg free), and the mucosal layer was scraped off using a hemocytometer coverslip. The crypt harboring intestinal basal lamina was minced, washed rigorously with PBSO and disintegrated using EDTA (2 mM, 30 min, on ice). Intestinal fragments were dissociated through continuous pipetting and addition of 10% FBS in PBSO, and passed through a cell strainer (70 μm). Isolated crypts were washed twice, embedded in matrigel and plated into 24-well cell culture plates. After solidification of the matrigel, complete growth medium for small intestine organoids was added (Advanced DMEM/F12 supplemented with GlutaMax, Pen/Strep, Hepes, N2 supplement, B27 supplement, n-Acetylcysteine, R-spondin, 100 ng/ml Noggin, 50 ng/ml mEGF). Intestinal organoids were passaged every 7 days using mechanical disruption (21-G needle) and the growth medium was changed every other day.

### Ricin toxicity in intestinal organoids

Murine intestinal organoids were passaged as described^35^, and treated with or without ricin at the indicated concentration after 3 days. After another 3 days, organoids were analyzed for overall survival using light and fluorescence microscopy. Control organoids (vehicle treated) developed well-defined luminal structures surrounded by an epithelial monolayer with budding crypt-like domains on the outside. Ricin treatment of WT organoids caused substantial cell death, a lack of integrity of the outer epithelial monolayer and a loss of distinctive crypt-like domains. This overall damage eventually led to rupture of the intestinal organoids. The number of intact, compact organoids after exposure to the toxin is determined and quantified, relative to vehicle-treated control structures of WT and mutant organoids.

### Fucose supplementation and inhibitors

ℒ-fucose (Sigma-Aldrich, F2252-5G) was used to reconstitute fucosylation in *Slc35c1*-deficient cells. Inhibition of fucosylation in HDF, HL-60, MDCK and VERO cells was achieved using 2F-Peracetyl-Fucose (Merck Chemicals and Life Science, 344827)^22^; murine organoids were treated with 2-deoxy-2-fluoro-ℒ-Fucose (Carbosynth, MD06089)^23^. Sialylation was inhibited using 3Fax-Peracetyl Neu5Ac (Merck Chemicals and Life Science, US1566224)^22^.

### Statistics

All values in the paper are given as means ± SD, unless stated otherwise. All experiments were reproduced 2-7 independent times. GraphPad Prism was used to generate figures and perform statistical analyses (GraphPad Software). An *a priori* sample size estimation was not performed. Data were analyzed by using the unpaired two-tailed Student's *t*-test, as indicated. *P* < 0.05 was accepted as statistically significant.

## Author Contributions

JT, JS and JMP were responsible for the design of the study. JT performed most *in vitro* and all *in vivo* experiments and data analysis. TIK and KS designed and performed the intracellular toxin trafficking assays. JS and KM helped with data analysis and conclusions. AL and UE provided expertise in haploid embryonic stem cell technology. Human tissue was obtained and provided by MJ, TM, CT and CK. CDR and JCP provided expertise in design of inhibitor experiments. JJ and JZ provided valuable advice in overall vector and sgRNA design. JT, JS and JMP wrote the manuscript with input from all authors.

## Competing Financial Interests

The authors declare no competing financial interests.

## Supplementary Information

Supplementary information, Figure S1 Schematic of intracellular fucosylation mediated by Slc35c1 and Fut9.

Supplementary information, Figure S2 *Slc35c1* and *Fut9* mutant embryonic stem cells lack fucosylation and exhibit increased resistance to ricin and RCA_120_.

Supplementary information, Figure S3 *Slc35c1* mutant cells show fucose-dependent resistance to ricin.

Supplementary information, Figure S4 Modulation of fucosylation and sialylation alter ricin susceptibility.

Supplementary information, Figure S5 St3Gal4 controls ricin susceptibility.
