# Supplementary material for: A vital sugar code for ricin toxicity
Source: Cell Res. 2017 Sep 19;27(11):1351–64. doi: 10.1038/cr.2017.116 (PMC5674155; doi:10.1038/cr.2017.116)
Supplement: Supplementary information, Figure S1 — Schematic of intracellular fucosylation mediated by Slc35c1 and Fut9. [file cr2017116x1.pdf]

Supplementary information, Figure S1

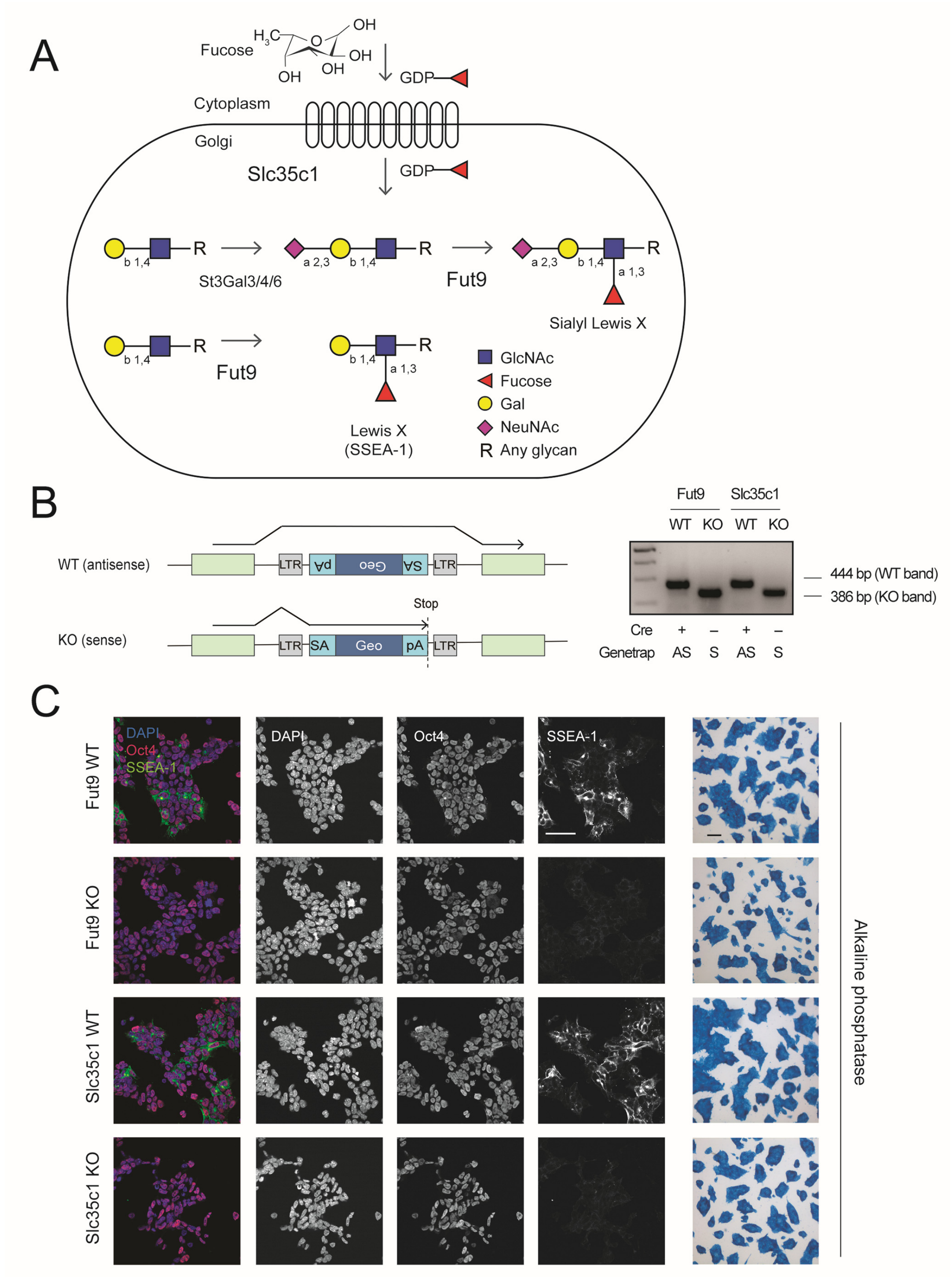

Figure S1 Schematic of intracellular fucosylation mediated by Slc35c1 and Fut9. **(A)** The GDP-fucose transporter Slc35c1 is expressed in the Golgi membrane and enables

uptake of GDP-fucose into the Golgi compartment. Different fucosyltransferases catalyze the attachment of activated GDP-fucose to acceptor carbohydrate structures of proteins in a cell. The α1,3 fucosyltransferase Fut9 is specifically required for the formation of the Lewis X and sialyl Lewis X epitopes. GlcNAc (N-acetylglucosamine), Gal (Galactose), NeuNAc (N-Acetylneuraminic acid, one type of sialic acid). **(B)** Haploid murine embryonic stem cells were mutagenized, using a gene trap insertional mutagenesis cassette in introns of either the *Fut9* or *Slc35c*1 genomic locus. Expression of Cre recombinase in *Fut9* or *Slc35c1* knockout (KO) cells (sense integration into an intron that affects splice-acceptors) leads to inversion of the mutagenic construct into anti-sense and reconstitution of wild-type (WT) gene expression. Genotyping PCR detects the mutagenesis cassette either in sense (KO, lower band) or anti-sense (WT, upper band). S, sense; AS; anti-sense; LTR, long terminal repeat; pA, polyA signal; Geo, b-Geo selection cassette. **(C)** *Fut9* and *Slc35c1* mutant mouse embryonic stem cells, as well as their WT sister cell clones were stained for the stemness markers Oct4, SSEA-1 and alkaline phosphatase. DAPI is shown as a nuclear counterstain. Cells were analyzed using fluorescence microcopy. Scale bar, 50 μm.
