# Supplementary material for: A vital sugar code for ricin toxicity
Source: Cell Res. 2017 Sep 19;27(11):1351–64. doi: 10.1038/cr.2017.116 (PMC5674155; doi:10.1038/cr.2017.116)
Supplement: Supplementary information, Figure S2 — Slc35c1 and Fut9 mutant embryonic stem cells lack fucosylation and exhibit increased resistance to ricin and RCA120. [file cr2017116x2.pdf]

Supplementary information, Figure S2

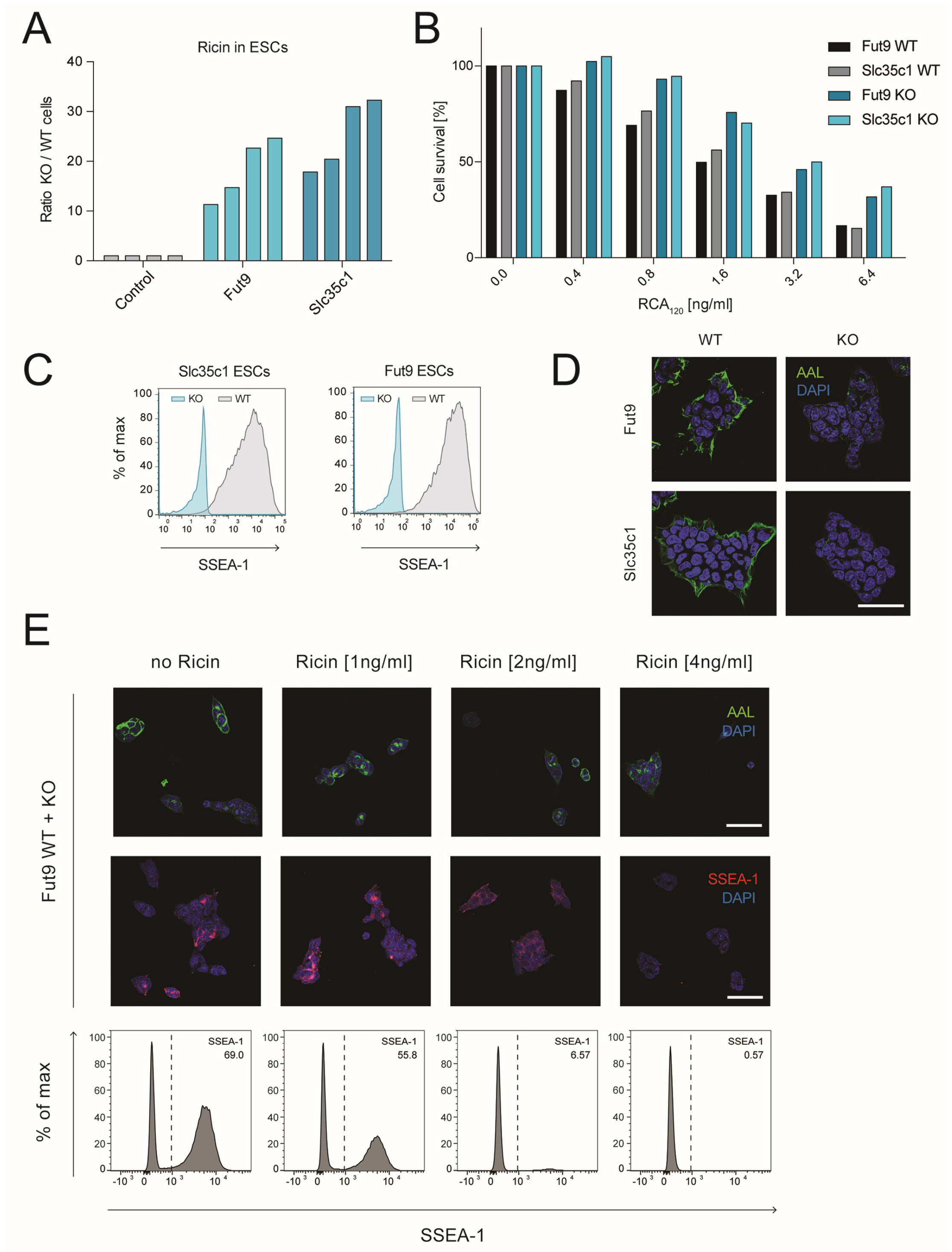

Figure S2 *Slc35c1* and *Fut9* mutant embryonic stem cells lack fucosylation and exhibit increased resistance to ricin and RCA_{120}. **(A)** Murine embryonic stem cells (mESCs)

harboring a reversible gene trap in *Fut9* or *Slc35c1*, as well as control cells, were infected with GFP and mCherry-Cre expressing retroviruses. Mixed populations of GFP and mCherry positive cells were subjected to ricin (2 ng/ml) for 10 days. The ratios of cells expressing GFP (KO) and mCherry (WT) were determined via FACS and values were normalized to untreated cells. Each bar represents an independent clonal cell line. **(B)** Survival of sense (KO) and anti-sense (WT) mESCs harboring gene traps in either *Fut9* or *Slc35c1* subjected to the indicated doses of RCA_{120}. Viability was determined using Alamar Blue. Representative data of two independent experiments are shown. **(C)** mESCs harboring gene traps in either sense (KO) or antisense (WT) orientation were stained for SSEA-1 (Lewis X, CD15) expression and analyzed with flow cytometry. Exemplary FACS histograms are shown. **(D)** mESCs harboring genetraps in either sense (KO) or antisense (WT) orientation were staining with AAL (*Aleuria aurantia* lectin) to detect the presence or absence of fucose-containing glycans. Cells were analyzed using fluorescence microcopy. DAPI is shown as a nuclear counterstain. Scale bar, 50 µm. **(E)** Mixed populations of unlabeled *Fut9* WT and KO sister mESCs were subjected to different concentrations of ricin for 3 days. The presence of fucose (*Aleuria aurantia* lectin, AAL) and Lewis X (SSEA-1, CD15) positive cells was determined using immunofluorescence microscopy (upper panels) and flow cytometry (lower panels). Scale bar, 50 µm.
