# Supplementary material for: A vital sugar code for ricin toxicity
Source: Cell Res. 2017 Sep 19;27(11):1351–64. doi: 10.1038/cr.2017.116 (PMC5674155; doi:10.1038/cr.2017.116)
Supplement: Supplementary information, Figure S3 — Slc35c1 mutant cells show fucose-dependent resistance to ricin. [file cr2017116x3.pdf]

Supplementary information, Figure S3

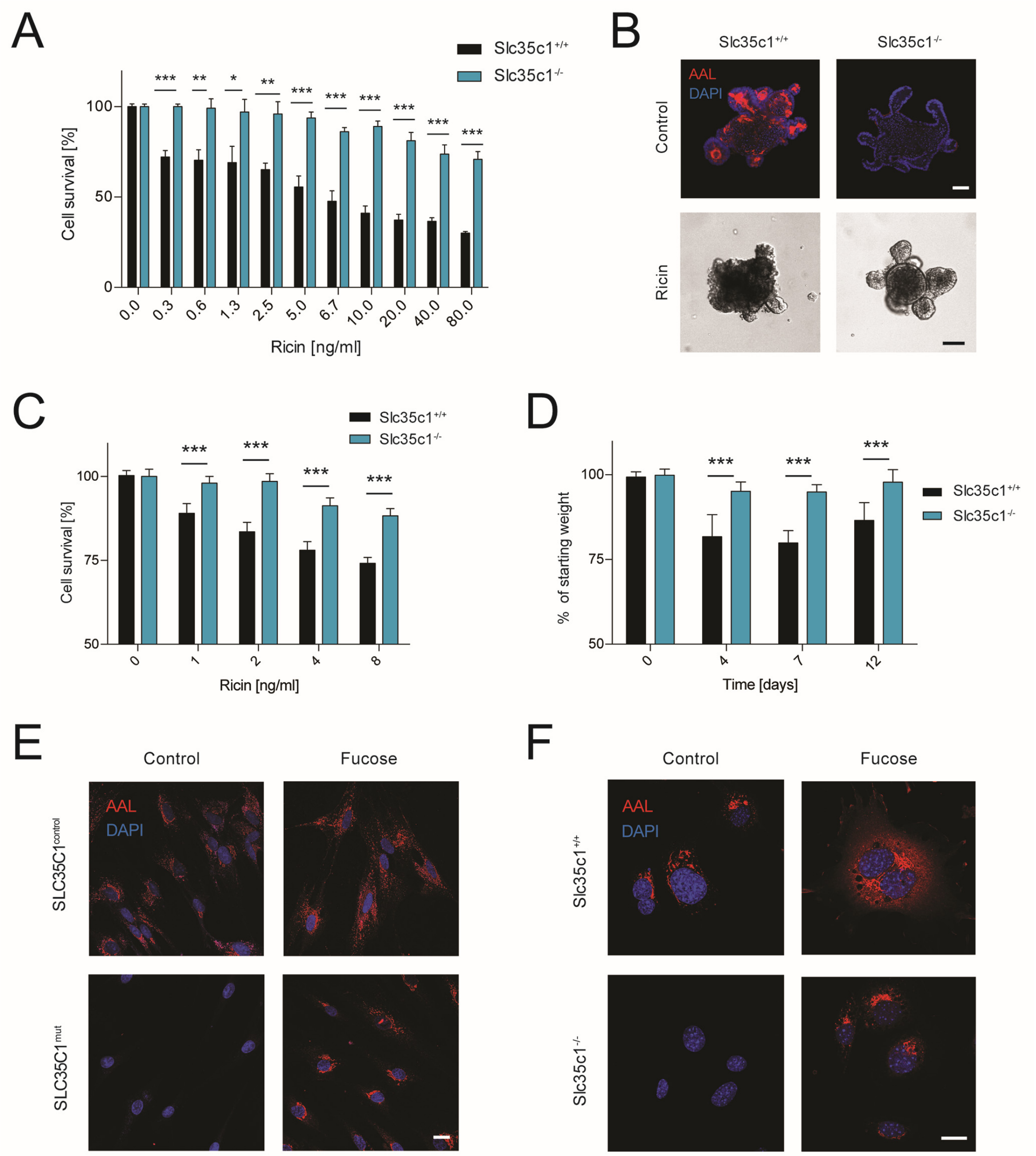

Figure S3 *Slc35c1* mutant cells show fucose-dependent resistance to ricin. **(A)** *Slc35c1* wild-type (*Slc35c1*$^{+/+}$) and knockout (*Slc35c1*$^{-/-}$) mouse embryonic fibroblasts (MEFs) were cultivated in the presence or absence of ricin for 48 h. Cell survival was determined using Alamar Blue cell viability assay. Data are shown as mean ± SD of triplicate cultures. The experiment was repeated four times. *$P < 0.05$, **$P < 0.01$, ***$P < 0.001$ (Student's *t*-

test). **(B)** Intestinal organoids derived from *Slc35c1*$^{-/-}$ and *Slc35c1*$^{+/+}$ mice were stained with AAL (top) and treated with ricin (8 ng/ml) or left untreated (control). Organoids were analyzed on day 5. Scale bar, 50 µm. **(C)** Splenocytes from *Slc35c1*$^{+/+}$ and *Slc35c1*$^{-/-}$ mice were isolated, stimulated and treated with different dosages of ricin. Viable cells were quantified via flow cytometry using eFluor780 viability dye. Data are shown as mean ± SD of quadruplicate cultures. The experiment was repeated twice. **(D)** *Slc35c1*$^{+/+}$ and *Slc35c1*$^{-/-}$ mice were treated intravenously with sub-lethal dosages of ricin (0.53 µg/kg) and their weight loss was monitored over time. n = 3 wild-type and n = 6 knockout mice per group. **(E)** Mutant *(Slc35c1*^{mut}) and control (*Slc35c1*^{control}) human dermal fibroblasts were supplemented with fucose (10 mM) for 24 h and stained with *AAL* to assess the presence or absence of fucose containing epitopes. Scale bar, 20 µm. **(F)** *Slc35c1*$^{+/+}$ and *Slc35c1*$^{-/-}$ MEFs were supplemented with fucose (10 mM) for 24 h and stained with AAL. Scale bar, 25 µm. *$P < 0.05$, **$P < 0.01$, ***$P < 0.001$ (Student's *t*-test).
