# Supplementary material for: A vital sugar code for ricin toxicity
Source: Cell Res. 2017 Sep 19;27(11):1351–64. doi: 10.1038/cr.2017.116 (PMC5674155; doi:10.1038/cr.2017.116)
Supplement: Supplementary information, Figure S4 — Modulation of fucosylation and sialylation alter ricin susceptibility. [file cr2017116x4.pdf]

Supplementary information, Figure S4

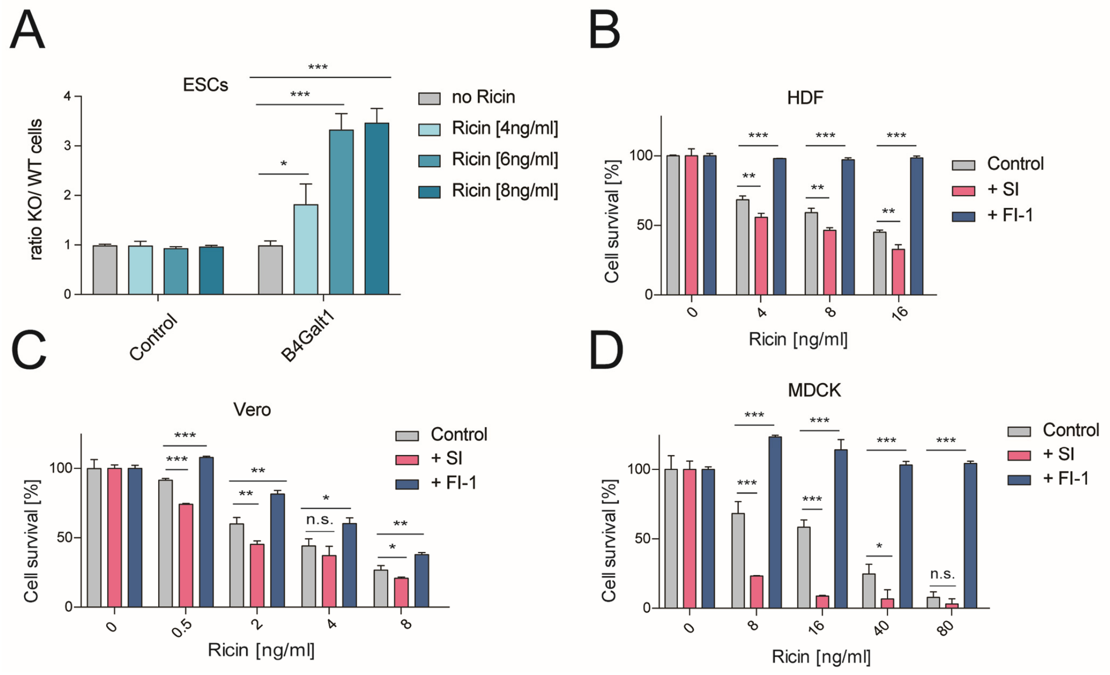

Figure S4 Modulation of fucosylation and sialylation alter ricin susceptibility. **(A)** Mixed populations of cells that harbor a reversible gene trap in B4Galt1 as well as wild-type control cells, were subjected to different doses of ricin for 3 days. The ratio of mutant (sense, GFP) to wild-type (antisense, mCherryCre) cells was assessed via flow cytometry. **(B)** Human dermal fibroblasts (HDF), **(C)** simian Vero cells and **(D)** Madin-Darby canine kidney (MDCK) cells were treated with inhibitors of fucosylation (FI-1, 100 µM) or sialylation (SI, 250 µM). The cells were then subjected to different concentrations of ricin for 2 days and their survival was determined using Alamar Blue. Data in **A**-**D** are shown as mean ± SD of triplicate cultures. The experiments were repeated at least three times. **P* < 0.05, ***P* < 0.01, ****P* < 0.001; n.s., not significant (Student's *t*-test).
