# Supplementary material for: A vital sugar code for ricin toxicity
Source: Cell Res. 2017 Sep 19;27(11):1351–64. doi: 10.1038/cr.2017.116 (PMC5674155; doi:10.1038/cr.2017.116)
Supplement: Supplementary information, Figure S5 — St3Gal4 controls ricin susceptibility. [file cr2017116x5.pdf]

Supplementary information, Figure S5

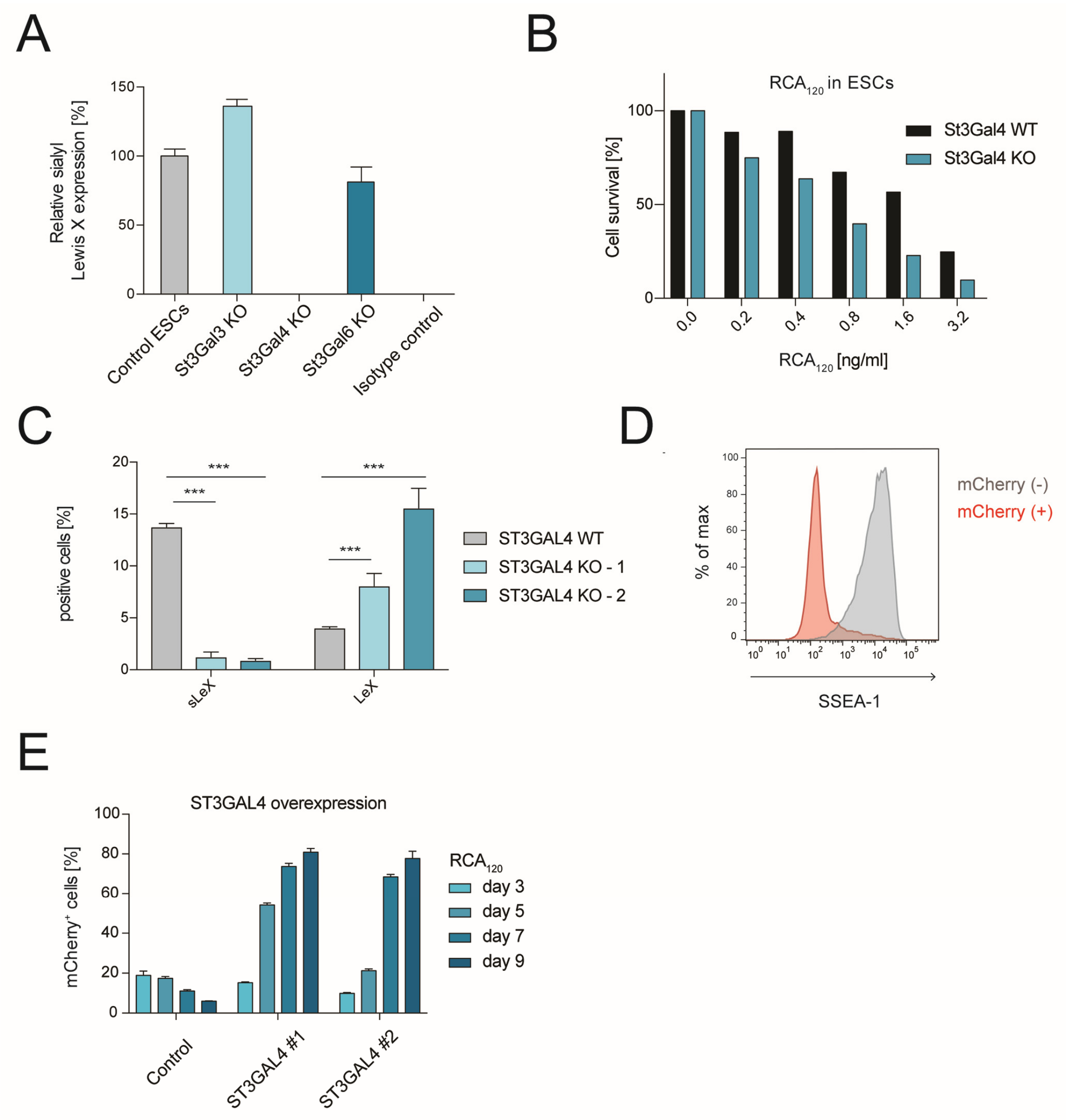

Figure S5 St3Gal4 controls ricin susceptibility. **(A)** Control cells, as well as *St3Gal*3, *St3Gal4* or *St3Gal6* mutant mouse ESCs were stained for sialyl Lewis X (CD15s). The amount of the sialic acid containing epitope was determined via FACS and normalized to control cells. Data are mean ± SD (n = 3). **(B)** *St3Gal4* knockout (KO, sense integration) and repaired wild type (WT, anti-sense) sister mESCs were exposed to RCA_{120} for 2 days

and their viability was determined using Alamar Blue. Representative data of three independent experiments are shown. **(C)** *ST3GAL4* control and mutant human KBM7 cells were stained for sialyl Lewis X (CD15s) as well as Lewis X (CD15) and analyzed via flow cytometry. Data are shown as mean ± SD of triplicate stainings. $*P < 0.05$, $**P < 0.01$, $***P < 0.001$; n.s., not significant (Student's *t*-test). Representative data of three independent experiments are shown. **(D)** Ricin-resistant, mCherry-positive, ST3GAL4-overexpressing cells, as well as control cells, were stained for SSEA-1 (CD15) and analyzed via flow cytometry. **(E)** Inducible, ST3GAL4-overexpressing cells were subjected to RCA_{120} (2 ng/ml) for 9 days and the amount of mCherry-positive cells was monitored by FACS at the indicated time points. Data are shown as mean ± SD of triplicate cultures.
